# The Role of High-Fidelity Team-Based Simulation in Acute Care Settings: A Systematic Review

**DOI:** 10.1055/s-0038-1667315

**Published:** 2018-08-13

**Authors:** Sarah Armenia, Loka Thangamathesvaran, Akia D. Caine, Neil King, Anastasia Kunac, Aziz M. Merchant

**Affiliations:** 1 Division of General Surgery, Department of Surgery, Rutgers University, New Jersey Medical School, Newark, New Jersey; 2 Division of Trauma and Surgical Critical Care, Department of Surgery, Rutgers University, New Jersey Medical School, Newark, New Jersey

**Keywords:** teaching, simulation training, team training, high fidelity simulation training, education, simulation-based medical education

## Abstract

**Introduction** High-fidelity team-based simulation has been identified as an effective way of teaching and evaluating both technical and nontechnical skills. Several studies have described the benefits of this modality in a variety of acute care settings, but a lack of standardized methodologies has resulted in heterogeneous findings. Few studies have characterized high fidelity simulation across a broad range of acute care settings and integrated the latest evidence on its educational and patient impact.

**Methods** The MEDLINE, EMBASE, Cochrane Library, and PsycINFO databases were searched for empirical studies from the last 10 years, investigating high fidelity team-based simulation in surgical, trauma, and critical care training curricula.

**Results** Seventeen studies were included. Interventions and evaluations were comprehensively characterized for each study and were discussed in the context of four overarching acute care settings: the emergency department/trauma bay, the operating room, the intensive care unit, and inpatient ad hoc resuscitation teams.

**Conclusions** The use of high-fidelity team-based simulation has expanded in acute care and is feasible and effective in a wide variety of specialized acute settings, including the emergency department/trauma bay, the operating room, the intensive care unit, and inpatient ad hoc resuscitation teams. Training programs have evolved to emphasize team-based, multidisciplinary education models and are often conducted in situ to maximize authenticity. In situ simulations have also provided the opportunity for system-level improvement and discussions of complex topics such as social hierarchy. There is limited evidence supporting the impact of simulation on patient outcomes, sustainability of simulation efforts, or cost-effectiveness of training programs. These areas warrant further research now that the scope of utilization across acute care settings has been characterized.

Teamwork and communication have been identified as critical components of safe healthcare systems. 1 Previous studies across several industries have recognized simulation as an effective way of improving these skills, particularly in the acute care setting where ad hoc teams form rapidly and require efficient collaboration. 2 3 The increasing complexity of simulation has enabled the assessment and development of technical and nontechnical skills in a diverse spectrum of acute care settings. 4 5 6 7 High-fidelity simulation specifically involves the use of a computerized full-body mannequin that can give dynamic, physiologic feedback and can be programmed to provide realistic responses. 8 This technology has facilitated several acute care team-based training programs and subsequently a growing body of research on their effectiveness. However, without standardized intervention and evaluation methodologies, the heterogeneity of these studies necessitates systematic analysis.

Previous reviews of the literature on simulation in acute care settings have focused on specific learning objectives, participant populations, or clinical environments within acute care. A review by Boling and Hardin-Pierce integrated research specifically on knowledge and confidence following high-fidelity simulation in critical care training. 5 In another review, Tan et al analyzed multidisciplinary team simulation specifically in the operating room. 9 Warren et al reviewed the effectiveness of simulation on satisfaction and learning outcomes in nurse practitioner programs. 10 Other reviews have separated their analysis by technical versus nontechnical skills. Gjerra et al and Lewis et al reviewed the impact of team-based simulation on nontechnical skills specifically. 3 4 A comprehensive analysis that appropriately reflects the breadth of participant populations, types of skills assessed, and scope of acute care settings is therefore necessary.

The purpose of this review was to synthesize the best available evidence on the utilization of high-fidelity team-based simulation in a broad scope of acute care settings. The goal was to explore the full scope of application of this modality to surgical, trauma, and critical care training curricula, to compare intervention and evaluation characteristics by acute care setting, and to integrate existing evidence from the last 10 years on actual patient outcomes. The research questions were as follows:

1. What is the scope of acute care settings in which high-fidelity team-based simulation is being utilized, and how do the characteristics of these simulations differ by setting?
2. How does in situ versus off site simulation study design compare in acute care team-based simulation training?
3. How does multidisciplinary team design impact the effectiveness of acute care team-based simulation training?
4. What translational progress has been made over the last 10 years in evaluating the impact of acute care simulation training on actual patient outcomes?

## Methods

### Search Strategy

**Fig. 1:**
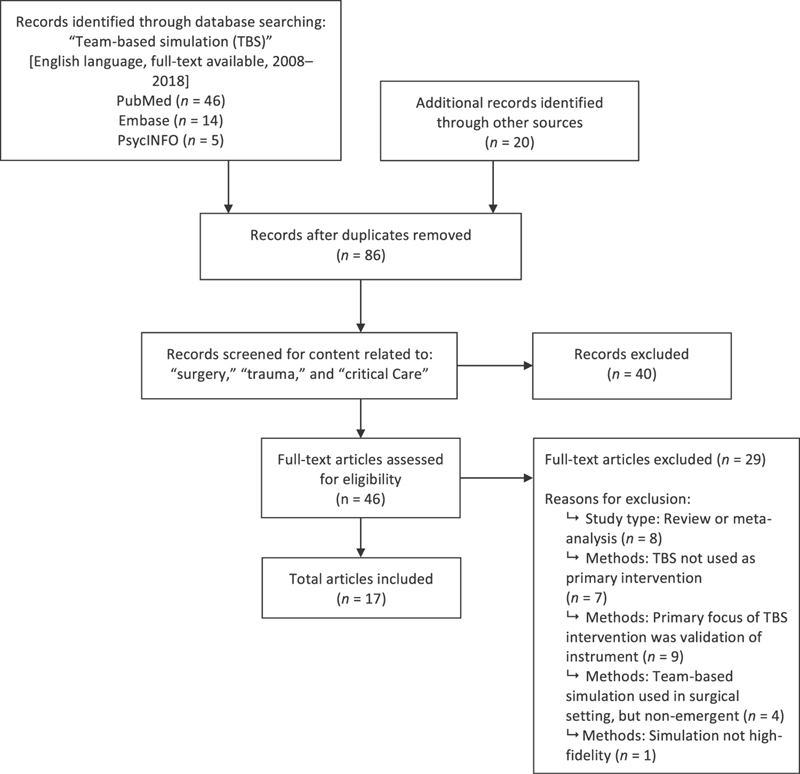
Flow diagram of article selection process.

An initial search of MEDLINE was conducted to identify index terms and keywords pertaining to “team-based simulation.” An extensive second search using all identified index terms and keywords was then performed in the following databases: MEDLINE, EMBASE, Cochrane Library, and PsycINFO. Keywords included “simulation,” “surgical procedures, operative,” “general surgery,” “trauma,” and “critical care.” Studies were limited to those in the English language with full text available. Due to the innovative and technology-driven nature of the subject, searches were limited to a 10-year period, including studies from January 1, 2008 to March 11, 2018 (date the search was performed). Finally, reference lists of all articles included thus far were searched for additional relevant citations. These articles were then imported to a reference management system for full-text review using pre-established inclusion and exclusion criteria ( Table 1 ). Since the objective of the search was to synthesize the available evidence regarding team-based simulation as a primary intervention, only empirical studies were included, and other types of studies, such as literature reviews or editorials, were excluded. A flow diagram illustrating the article selection process is included ( Fig. 1 ).

**Table 1:** Inclusion and exclusion criteria

Inclusion criteria	Exclusion criteria
▪ Peer-reviewed papers ▪ Published from 2008 to 2018 ▪ Published in any country ▪ Published in English ▪ Empirical studies that investigate technical and non-technical skills via team-based simulation training ▪ Acute care setting (emergency department/trauma bay; operating room; intensive care units; and ad hoc resuscitation/code teams).	▪ Incomplete reports (only abstract available; conference proceedings) ▪ Review articles ▪ Studies where instrument design and/or validation was the primary endpoint.

### Assessment of Quality

The articles were then assessed by two independent reviewers, the first author (Sarah Armenia) and second author (Loka Thangamathesvaran), using the Critical Appraisal Skills Program (CASP) to standardize the assessment process. 11 CASP is a 10-question checklist used to evaluate research studies and offers a systematic way to critically evaluate methodology across independent reviewers. Screening reliability between the two reviewers was assessed using Cohen's kappa at the abstract and full-text levels. 12 Any discrepancies were resolved by discussion with other co-authors, and the final consensus was confirmed by the senior author (Aziz M. Merchant).

### Data Synthesis

**Fig. 2:**
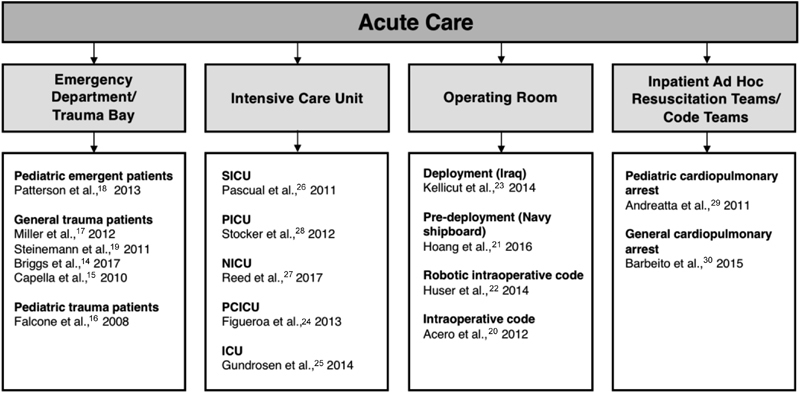
The spectrum of acute care settings where high-fidelity simulation is feasible for team-based training and further categorization of study populations and/or clinical contexts. Abbreviations: SICU, surgical intensive care unit; PICU, pediatric intensive care unit; NICU, neonatal intensive care unit; PCICU, pediatric cardiac intensive care unit; ICU intensive care unit.

After thematic analysis, four distinct clinical environment subtypes were identified under the umbrella of acute care: emergency department/trauma bay, operating room, intensive care unit, and inpatient ad hoc resuscitation/code teams. Information for setting subtype categorization was found in either the objective, description of the study setting and participants, or the methodology for studies with in situ interventions. In studies that were conducted offsite, setting subtype information was found in the objective or the methodology (which included descriptions of the specific environment that the intervention was attempting to simulate). Two studies with in situ interventions did not simulate a fixed clinical environment for simulations and were therefore assigned to a separate category. These studies designed unannounced simulations that were triggered at various locations throughout the medical center resulting in the formation of ad hoc teams sent to various inpatient settings. Studies categorized into these four acute care setting subtypes were then further characterized by distinguishing features of their simulated patient populations (i.e., pediatric trauma patients) or their study participants (i.e., military personnel deployed in Iraq) as seen in Fig. 2 .

For each acute care setting, characteristics of the intervention and evaluation were tabulated systematically. Each intervention was characterized by using the simulation technology, whether it was conducted in situ or off site, and the scope of clinical scenario(s) simulated ( Table 2 ). Each evaluation was characterized by the type of skill assessed (technical, nontechnical, or both), the Kirkpatrick's level(s) of evaluation ( Table 3 ), the simulation scoring instrument, and the debriefing process ( Table 4 ). The Kirkpatrick model of evaluation has been used previously to assess evidence in educational research and provides a systematic way of categorizing learning outcomes. 13 In this model, Kirkpatrick Level 1 evaluates participant satisfaction; Level 2 evaluates knowledge acquisition; Level 3 evaluates participant behavior change; and Level 4 evaluates improved patient outcomes. It is possible for an evaluation to cover multiple Kirkpatrick's levels of evaluation as seen in Table 3 .

**Table 2:** Characteristics of the interventions: simulation technology, context, and scenario(s)

Acute care setting	Sources	Simulation technology	In situ	Description of clinical scenario(s)
Emergency department/trauma bay	Briggs et al., 2017	High-fidelity trauma simulation (STRATUS Center for Surgical Simulation)		Blunt trauma from a motor vehicle accident; multiple penetrating injuries from a broken-plate glass window
	Capella et al 15	High-fidelity trauma simulation (Carilion Clinic Center for Experiential Learning)		Unstable patient after motor vehicle accident with penetrating injuries; similar scenarios (referred to but not provided)
	Falcone et al 16	SimBaby™, PediaSIM, and SimMan® (Medical Educational Technologies Incorporated, Sarasota, Fla)		Infant with head injury; Child with a penetrating wound to the back; adolescent with multitrauma including an unstable pelvic fracture
	Miller et al 17	SimMan® 3G (Laerdal, Stavanger, Norway)	♦	Patient with blunt abdominal trauma in obvious shock with an intact airway and a FAST examination positive for intraperitoneal free fluid; patient with penetrating chest injury who arrived without intravenous access and required advanced airway management, tube thoracostomy, and pericardiocentesis for stabilization
	Patterson et al 18	High-fidelity simulator (not specified)	♦	Trauma and medical simulations based on high-risk clinical cases (not specified)
	Steinemann et al 19	SimMan® 3G (Laerdal, Stavanger, Norway)	♦	Three preprogrammed, β-tested, 15-min blunt traumatic shock scenarios (not specified)
Operating room	Acero et al 20	Simulated OR equipped with a SimMan® 3G (Laerdal, Stavanger, Norway)		Pregnant simulated patient in hemorrhagic shock, bleeding from a carotid injury, ultimately leading to cardiac arrest
	Hoang et al 21	Human-Worn, Partial-Task, Surgical Simulator, also known as the “Cut Suit” (Strategic Operations Inc., San Diego, CA); Cadavers	♦	Simulated trauma involving one or two casualties per scenario every day, with increasing complexity, based on concepts taught earlier that day; final 6-h mass casualty event on the last day
	Huser et al 22	METI iSTAN (CAE Healthcare, Sarasota, FL) modified to hold five trocars	♦	Ventricular fibrillation in a simulated patient docked to a robot
	Kellicut et al 23	High-fidelity simulator (not specified); medical moulage application to simulate casualties	♦	Twenty trauma scenarios were created from the Baghdad Combat Support Hospital trauma patient database
Intensive care units (adult, pediatric; cardiac, and surgical)	Figueroa et al 24	Newborn HAL®, Pediatric HAL® (Gaumard, Miami, FL); SimMan® (Laerdal, Stavanger, Norway)		Postoperative Norwood patient with accidental extubation; Glenn patient with postoperative hemorrhagic stroke; Fontan patient with low cardiac output; BT shunt with acute thrombosis; postoperative Ebstein's repair with unstable SVT; postoperative TOF patient with low cardiac output syndrome; and jet ventilation leading to ECMO
	Gundrosen et al 25	SimMan® 2G (Laerdal, Stavanger, Norway)	♦	Septic shock
	Pascual et al 26	SimMan® (male and female) with technical manipulation using SimMan Software version 3.3.1 (Laerdal, Stavanger, Norway)		Anaphylaxis with tension pneumothorax; Septic shock from *Clostridium difficile* colitis; Myocardial infarction with diabetic ketoacidosis; hemorrhagic shock with abdominal compartment syndrome; deteriorating traumatic brain injury with status epilepticus
	Reed et al 27	Baby Anne® manikin used for low-fidelity simulation (Laerdal, Stavanger, Norway); Premie, Newborn and Pediatric HAL® simulators used for high-fidelity simulation (Gaumard, Miami, FL)	♦	Home birth (esophageal intubation, hypothermia, hypoglycemia); respiratory failure (sepsis/pneumonia); cardiogenic shock; ECPR (cardiac, general surgery); post sternotomy chest compressions; tracheostomy dislodgment; ventricular fibrillation; supraventricular tachycardia; acute pulmonary hemorrhage; tension pneumothorax; pneumothorax postsurfactant; myelomeningocele self-extubation; Premie self-extubation; fentanyl-induced rigid chest; and apnea/bradycardia/desaturation event
	Stocker et al 28	SimBaby™ (Laerdal, Stavanger, Norway)	♦	Respiratory problems (respiratory arrest, blocked endotracheal tube, pneumothorax, and severe asthma exacerbation); cardiac problems (cardiac arrest, pericardial effusion, thrombosed arterio-pulmonary shunt, post-operative low cardiac output state, and post-surgical cardiac tamponade); and general PICU problems (hyperkalemic rhythm disturbance and supraventricular tachycardia)
Inpatient ad hoc resuscitation teams/code teams	Andreatta et al 29	METI PediaSIM (CAE Healthcare, Sarasota, FL); SimBaby™ (Laerdal, Stavanger, Norway)	♦	Sepsis (immunosuppressed and normal patients); respiratory distress (bronchiolitis, pneumonia); increased intracranial pressure/herniation (intracranial mass, intracranial trauma, and meningitis, seizures); anaphylactic shock; and cardiogenic shock (congestive heart failure, congenital heart disease, and myocarditis)
	Barbeito et al 30	SimMan® 3G (Laerdal, Stavanger, Norway)	♦	Cardiac arrest

Abbreviations: BT, Blalock–Thomas–Taussig; ECMO, extracorporeal membrane oxygenation; ECPR, extracorporeal cardiopulmonary resuscitation; FAST, focused assessment with sonography for trauma; PICU, pediatric intensive care unit; STRATUS, simulation, training, research and technology utilization system; SVT, supraventricular tachycardia; TOF, tetralogy of fallot.

**Table 3:** Characteristics of the evaluation of effect of each simulation intervention by type(s) of skills evaluated and the four Kirkpatrick levels of evaluation a for each acute care setting

Acute care setting	Sources	Technical skills	Nontechnical skills	Both	Kirkpatrick's levels of evaluation b			
					Reaction	Learning	Behavior	Outcomes
Emergency department/trauma bay	Briggs et al., 2017			♦		♦		
	Capella et al 15			♦			♦	♦
	Falcone et al 16	♦			♦	♦		
	Miller et al 17		♦		♦		♦	
	Patterson et al 18			♦	♦			
	Steinemann et al 19			♦		♦	♦	♦
Operating room	Acero et al 20	♦			♦	♦		
	Hoang et al 21	♦				♦		
	Huser et al 22	♦				♦		
	Kellicut et al 23			♦	♦	♦		
Intensive care units (adult, pediatric; cardiac, surgical)	Figueroa et al 24			♦		♦		
	Gundrosen et al 25			♦		♦		
	Pascual et al 26			♦	♦	♦		
	Reed et al 27	♦			♦	♦	♦	
	Stocker et al 28			♦	♦	♦		
Inpatient ad hoc resuscitation teams/code teams	Andreatta et al 29	♦			♦	♦	♦	♦
	Barbeito et al 30			♦			♦	♦

a Adapted from Kirkpatrick. 13

b Level 1: Reaction (participant satisfaction), Level 2: learning (knowledge, skills and attitudes), Level 3: behavior (translation of learning to clinical setting), and Level 4: outcome (patient outcomes).

**Table 4:** Characteristics of the instrument used to score simulation interventions (including whether it is validated a ) and the debriefing process following the simulation

Acute care setting	Sources	Simulation scoring instrument		Debriefing process
		Technical skills	Nontechnical skills	
Emergency department/Trauma bay	Briggs et al 14	Clinical checklist; times to specific task completion	NOTSS a ; T-NOTECHS a	None (retrospective study design)
	Capella et al 15	N/A; Resuscitations pre- and post-training were scored, not simulations	N/A; resuscitations pre- and post-training were scored, not simulations	Videotapes (of resuscitations pre- and post-training) reviewed immediately after simulation
	Falcone et al 16	Instrument developed by Holcomb et al., 2001	Not assessed	Videotapes reviewed immediately after simulation
	Miller et al 17	Not assessed	CTS a	Immediately after simulation; Focused on teamwork
	Patterson et al 18	Not scored; concepts discussed in debriefing	Modified ANTS a	Immediately after simulation; focused on teamwork and system-level safety threats
	Steinemann et al 19	Clinical process parameters checklist	T-NOTECHS a	Videotapes reviewed immediately after simulation; focused on teamwork
Operating room	Acero et al 20	Number of mitigation steps completed; indirectly assessed through questionnaire (testing clinical knowledge)	Not assessed	Videotapes reviewed immediately after both “cold” and “warm” simulations
	Hoang et al 21	Disposition time and critical errors made (assessed at three time points for comparison)	Not assessed	None
	Huser et al 22	Times to specific task completion	Not assessed	Same day as simulation; Focused on teamwork and system-level safety threats
	Kellicut et al 23	Prehospital, triage, and resuscitation evaluation checklists	Component of triage and resuscitation evaluation checklists	Videotapes reviewed immediately after simulation; Focused on teamwork
Intensive care units (adult, pediatric; cardiac, and surgical)	Figueroa et al 24	Clinical process parameters checklist	Principles of Team STEPPS assessed	Immediately after simulation
	Gundrosen et al 25	Clinical checklist; times to specific task completion	ANTS a	Videotapes reviewed immediately after simulation
	Pascual et al 26	ECCS; indirectly assessed through written examination pre- and post-course	TLIS	Videotapes reviewed immediately after simulation
	Reed et al 27	Clinical checklist	Not assessed	Immediately after simulation; Individual, equipment and system-level issues
	Stocker et al 28	Not scored; Assessed through self-evaluation	Not scored; assessed through self-evaluation	Based on the Children's Hospital Boston Simulation Program teaching principles of crisis resource management
Inpatient ad hoc resuscitation teams/code teams	Andreatta et al 29	Not scored; assessed through self-evaluation and indirectly through survival rates longitudinally	Not scored; assessed through self-evaluation	Videotapes reviewed immediately after simulation
	Barbeito et al 30	Not scored; Concepts discussed in debriefing	Not scored; concepts discussed in debriefing	Videotapes reviewed immediately after simulation; focused on teamwork and system-level threats

Abbreviations: ANTS, Anesthetists' Non-Technical Skills; CTS, clinical teamwork scale; ECCS, emergency clinical care skills; NOTSS, non-technical skills for surgeons; Team STEPPS, Team Strategies and Tools to Enhance Performance; TLIS, Team Leadership-Interpersonal Skills; T-NOTECHS, modified non-technical skills scale for trauma.

a Indicates the instrument has been validated.

## Results

### Overview of the Included Studies

Seventeen studies met the inclusion criteria. The studies originated from four countries: the United States ( *n* = 14), Norway ( *n* = 1), England ( *n* = 1), and Germany ( *n* = 1). The types of journals covered a broad spectrum of disciplines: surgical education ( *n* = 5), surgery ( *n* = 2), endourology ( *n* = 1), pediatric cardiology ( *n* = 1), intensive and critical care nursing ( *n* = 1), intensive and critical care medicine ( *n* = 1), perinatology ( *n* = 1), pediatric critical care ( *n* = 1), simulation in healthcare ( *n* = 1), emergency medicine ( *n* = 1), trauma ( *n* = 1), and quality and safety ( *n* = 1). Journal quality was assessed using the SCImago Journal Rank (SJR Indicator), which is based on the number of citations received by a journal and the quality of the journals those citations came from. The studies were categorized into four acute care setting subtypes: emergency departments/trauma bays ( *n* = 6), operating rooms ( *n* = 4), intensive care units ( *n* = 5), and inpatient ad hoc resuscitation teams ( *n* = 2). Six studies assessed only technical skills, 1 study assessed only nontechnical skills, and 10 studies assessed both. Five of the studies used validated instruments for these assessments. Eleven studies implemented their simulations in situ, and six studies conducted the simulations in offsite simulation centers. Fifteen studies had multidisciplinary participants; one study consisted of only nurses; and one study consisted of only advanced practitioners. Evaluation was done at several Kirkpatrick levels—the effect on learning (Level 2) was most frequently evaluated (13 of 17 studies) followed by the effect on reaction (Level 1), consisting of 9 of 17 studies.

### Characteristics of Emergency Department/Trauma Bay Simulations

Six studies developed training programs that simulated a crisis within the emergency department or trauma bay setting. 14 15 16 17 18 19 One study simulated emergent care in the pediatric population; 18 one study simulated trauma in the pediatric population; 16 and the remaining four studies simulated trauma in adult populations. 14 15 17 19 Study design was heterogeneous with a wide variety of outcome measures. One study used simulation as a means of identifying latent safety threats and made changes at the system level. 18 One study assessed sustainability, observing that the scored behaviors returned to baseline after simulations stopped. 17 Most studies evaluated simulations, but Miller et al, Steinemann et al, and Capella et al observed actual trauma resuscitations as part of the study design. 15 17 19 One study assessed technical skills only; 16 another assessed nontechnical skills only, 17 and the remaining four studies assessed both skillsets. 14 15 18 19 Evaluation was done at several Kirkpatrick levels—notably, two of the four studies included in this review that evaluated the effect on outcomes (Level 4) were from this acute care setting subgroup. 15 19

### Characteristics of Operating Room Simulations

Four studies developed training programs that simulated a crisis requiring operative care as part of the intervention. 20 21 22 23 One study simulated an operative emergency occurring during active deployment in Iraq. 23 One study simulated an operative emergency on a Navy Shipboard as a part of pre-deployment training. 21 The remaining two studies developed interventions that simulated crises occurring during ongoing operations. 20 22 One study simulated an intraoperative code during general surgery, 20 and another study simulated an intraoperative code during robotic surgery, while the robot was actively docked. 22 A summary of simulations in this setting is provided in Table 5 . Three studies included a didactic and simulation component, while one study included only a simulation component. 20 21 23 One of the four studies evaluated the sustainability of the training after 5 months. 21 The operating room subgroup had the largest proportion of studies that assessed technical skills only (three of four studies). 20 21 22 The remaining study assessed both skillsets. 23 This emphasis on technical skills was reflected in the evaluations—all four studies used clinical checklists and times to task completion. Nontechnical skills were evaluated as part of a larger checklist in one study. 23 Evaluation was done at several Kirkpatrick levels —all four studies evaluated the effect of simulation on the learning level (Level 2), and two studies also evaluated the effect on the reaction level (Level 1). 20 23 A summary of evaluation characteristics can be found in Tables 3 and 4 . One study addressed system-level issues—the simulation of resuscitation during robotic surgery prompted the formation of a flow diagram by a multidisciplinary team after the first simulation. 22 This flow diagram contributed to better outcomes in the second simulation.

**Table 5:** Summary of included studies

Study	Study design	Participants	Objective	Intervention	Outcome measures	Results	SJR indicator a
Acero et al 20	Pre-/post-intervention study Single site	171 OR staff members (surgery residents, anesthesia residents, perioperative nurses)	Evaluate whether a high-fidelity mannequin improves team performance in a high-risk surgical emergency	Exsanguination scenario using high-fidelity mannequin	Team performance of eight mitigation steps at baseline (“cold”) vs debriefing and didactic session (“warm”)	Team training using high-fidelity simulation is effective in training OR staff in a high-risk surgical emergency	0.983
Andreatta et al., 2016	Longitudinal, mixed-methods Single site	252 resident encounters (some redundancy)	Evaluate viability and effectiveness of a simulation-based pediatric mock code program on patient outcomes; evaluate residents' confidence in performing resuscitations	Mock pediatric codes at increasing rates over a 48-month period	Self-assessment data; hospital records for pediatric CPA survival rates throughout study duration	Survival rates increased to approx. 50% correlating with the increased number of mock codes and remained stable for 3 years	1.359
Barbeito et al 30	Post-intervention study Single site	>300 (87 physicians, 100 nurses, 21 respiratory therapists, 10 administrative staff, remainder unspecified)	Identify opportunities for system optimization using an in situ simulation-based quality improvement program	Simulated unannounced cardiac arrest sessions	Technical aspects of session; structural and systems based hazards and defects	In situ simulation can identify and mitigate latent hazards and defects in the hospital emergency response system	0.567
Briggs et al 14	Retrospective cohort study	20 teams (surgical and emergency room residents; emergency department nurses; emergency services assistants)	Evaluate the effects of team leaders' nontechnical skills on technical performance of clinical tasks using simulated scenarios	Two separate high-fidelity, simulated trauma scenarios	Nontechnical skills (such as communication, leadership and teamwork) using the Modified Nontechnical Skills Scale for Trauma system	Nontechnical skills of trauma teams and trauma leaders deteriorate as clinical scenarios progress	0.983
Capella et all 15	Pre-/post-intervention study	28 surgery residents, 6 faculty surgeons, 80 emergency department nurses	Evaluate if formal team training improves team behaviors in trauma resuscitation; evaluate if this improvement increases efficiency and improves clinical outcomes	Didactic sessions; Multidisciplinary simulation sessions	Teamwork domain ratings (leadership, situation monitoring, mutual support and communication); time to definitive management	Structured trauma resuscitation team training augmented by simulation improves team performance	0.983
Falcone et al 16	Longitudinal; pre-/post-intervention study Single site	160 (pediatric surgeons, emergency medicine physicians, surgery/pediatric residents, nurses, critical care fellows, paramedic, respiratory therapists)	Evaluate the impact of multidisciplinary simulation training in pediatric trauma team performance	Monthly high-fidelity trauma simulations over 1-year period	Scoring tool assessing number of completed tasks in four areas: airway management, breathing, circulation and disability	Skills related to airway management, initial trauma assessment, cervical spine precautions and pelvic fracture recognition and management improved after team training	1.026
Figueroa et al., 2012	Pre-/post-intervention study Single site	37 (residents, 23 nurses, 5 respiratory therapists)	Evaluate whether a previously validated teamwork system using simulation-based team training (SBTT) would help improve perception of teamwork, confidence, and communication during post pediatric cardiac surgery cardiac arrest	Six simulated post-pediatric cardiac surgery scenarios (airway, neurologic and cardiac emergencies)	Surveys performed before, immediately after, and 3 months after participation	SBTT is effective in improving communication and increasing confidence among members of a multidisciplinary team during crisis scenarios	0.787
Gundrosen et al 25	Pre-/post-intervention study Single site	72 nurses	Evaluate the use of in situ simulation to explore team competence of ICU nurses	Participants randomized to either lecture-based or simulation-based teaching of septic shock in the ICU	“Team working” and “situation awareness” evaluated by two blinded raters	In situ simulation may be feasible for assessing competence in ICUs; No statistically significant difference between learning groups	0.564
Hoang et al 21	Prospective observational study	US Navy medical personnel (deployed physicians, corpsmen, nurses, nurse anesthetists)	Evaluate the ability of a simulation-based training course to produce sustained improvement in teamwork, communication, knowledge and trauma management; decrease time needed to complete tasks; decrease errors	Simulated trauma using a Human-Worn Partial-Task Surgical Simulator and cadavers	Time to disposition and critical errors made during simulation	Course demonstrated sustained improvement; can improve trauma care provided by Navy medical personnel to wounded service members	0.983
Huser et al 22	Post-intervention study Single site	18 (nurses, anesthesiologists, urologists, gynecologists)	Evaluate acute emergency management in an OR during a robotic-assisted surgery of a human simulator	Simulated emergency during robotic-assisted surgery of a human simulator	Time to start of chest compressions, removal of robotic system, first defibrillation and stabilization of circulation	Problems that arose during the first emergency simulation were solved and improvements were noted during repetition of simulation after debriefing	1.089
Kellicut et al 23	Post-intervention study	220 deployed personnel (physicians, nurse anesthetists, physician assistants, nurses, medics, OR technicians, other medical support personnel)	Evaluate a new educational and team-training program in a combat theater and assess staff perception following training	Simulation training models performed in the field (Iraq)	Anonymous surveys completed post-training	Surgical Team Assessment can be successfully implemented in an austere, hostile environment by incorporating simulation training models and TeamSTEPPs® concepts	1.174
Miller et al 17	Pre-/post-intervention study Single site	39 multidisciplinary teams (trauma surgeons and residents; ED physicians and residents; ED nurses; technicians; pharmacists; clerks; respiratory therapists	Evaluate whether an in situ trauma simulation program could be implemented and whether this would improve teamwork and communication	Weekly trauma simulations for 8 weeks	Clinical Teamwork Scale (CTS) was used to compare previously observed trauma activations to those activations during either a didactic-only period or simulation-only period	Improvements were noticed in all component measures during the in situ simulation intervention phase but this observed benefit declined after the simulation program stopped	1.593
Pascual et al 26	Pre-/post-intervention study Single site	12 advanced practitioners (APs)	Evaluate whether human patient simulator-based training is useful in established ICU APs	Five scenarios using a human patient simulator (mixed leader and observer roles)	Emergency care skills (airway-breathing-circulation sequence; recognition of shock; pneumothorax, etc.)	Human patient simulator training in established surgical ICU APs improves leadership, teamwork, and self-confidence skills in managing medical emergencies	N/A
Patterson et al 18	Post-intervention study Single site	218 healthcare providers	Identify latent safety threats at a higher rate than laboratory-based training; Reinforce teamwork training in a pediatric ED	90 in situ simulations of critical patients conducted over 1 year	Observed latent safety events (such as malfunctioning equipment or knowledge gaps); blinded video review using a modified Anesthetists' Non-Technical Skills scale to assess team behaviors	In situ simulation is a practical method for detection of latent safety threats and to reinforce training behaviors	2.54
Reed et al 27	Post-intervention study Single site	>500 NICU staff (neonatal/cardiac/surgical attendings; neonatal fellows; neonatal nurse practitioners; pediatric residents; nurses; respiratory therapists)	Evaluate team-based simulation training in the NICU setting	High- and low-fidelity simulation in the NICU (18 case scenarios) conducted for a 4-year period	Qualitative identification of systems issues and other areas needing improvement	Team-based simulation training is feasible and realistic in a busy NICU with appropriate planning and implementation.	0.906
Steinemann et al 19	Pre- post-intervention study Single site	137 team members (surgeons; emergency physicians; residents; physician assistants; nurses; respiratory therapists; emergency department technicians)	Evaluate impact of an HPS-based, in situ team training course on team communication, coordination, and clinical efficacy of trauma resuscitation	4-h HPS-based curriculum (web-based didactic followed by HPS training in emergency department)	Performance changes during HPS-based and actual trauma resuscitations	Improvement in teamwork ratings and clinical task speed and completion rates	0.983
Stocker et al 28	Pre-post intervention study Single site	219 PICU providers (nurses; cardiologists; intensivists; anesthetists; surgeons; allied health professionals)	Evaluate the impact of an embedded simulation-based team training program on perceived performance; Evaluate the effect over different phases of the program	3 phase program of simulated critical events over 2 years	Evaluation questionnaire (assessing impact on teamwork, communication skills, assessment skills, specific technical skills, confidence)	There is a 6- to 12-month learning curve in the implementation of an embedded multidisciplinary team training program; repeated exposure to simulation is most beneficial to crisis resource management training versus a single isolated exposure	0.692

Abbreviations: CPA, cardiopulmonary arrest; CRM, crisis resource management; ECMO, extracorporeal membrane oxygenation; ED, emergency department; HPS, human patient simulated; ICU, intensive care unit; NICU, neonatal intensive care unit; OR, operating room; PICU, pediatric intensive care unit; SBTT, simulation-based team training; SJR, SCImago Journal Rank; Team STEPPS®, Team Strategies and Tools to Enhance Performance and Patient Safety.

a SJR (SJR indicator) is a measure of scientific influence of scholarly journals that accounts for both the number of citations received by a journal and the importance or prestige of the journals where such citations come from. All ranks are from 2016 (most recent data available).

### Characteristics of Intensive Care Unit Simulations

Five studies developed training programs that simulated a crisis within intensive care units of various subspecialties. 24 25 26 27 28 The studies simulated a general intensive care unit, 25 surgical intensive care unit, 26 pediatric intensive care unit, 28 neonatal intensive care unit, 27 and a pediatric cardiac intensive care unit, 24 respectively. A summary of simulations in this setting is provided in Table 5 . Study design was heterogeneous—one study used pre- and post-intervention evaluations, 26 and one study use a randomization process to compare a didactic versus simulation-based curriculum. 25 Four studies assessed both technical and nontechnical skills, 24 25 26 28 and one study assessed only technical skills. 27 The evaluation tools for these parameters varied—several studies used a clinical process checklist for technical skills, 24 25 27 whereas one study used a previously validated score sheet developed by the National Registry of Emergency Medical Technicians. 26 Nontechnical skills were also evaluated in a variety of ways—one study assessed these skills through participant self-evaluation; 28 one study used Anesthetists' Non-Technical Skills (ANTS); 25 one study used Team Leadership-Interpersonal Skills (TLIS); 26 and Figueroa et al assessed principles of Team Strategies and Tools to Enhance Performance (Team STEPPs). 24 Evaluation was done at several Kirkpatrick levels. All five studies evaluated the effect of simulation on the learning level (Level 2); three studies also evaluated on the reaction level (Level 1); 26 27 28 and one study also evaluated on the behavior level (Level 3). 27 Reed et al evaluated changes in behavior qualitatively and indirectly, associating a decrease in the number of full codes following simulation with an increased aptitude for managing decompensating patients earlier in the process. 27 One study addressed system-level issues and conducted corresponding quality improvement efforts throughout the simulation period. 27 A summary of evaluation characteristics can be found in Tables 3 and 4 .

### Characteristics of Inpatient Ad Hoc Resuscitation Team/Code Team Simulations

Two studies developed training programs to simulate crises that would trigger the response of hospital-wide ad hoc resuscitation teams/code teams to an inpatient unit. 29 30 One study simulated a pediatric cardiopulmonary arrest (CPA), 29 and the other study simulated an adult CPA. 30 A summary of simulations in this setting is provided in Table 5 . One study evaluated only technical skills, 29 while the other study evaluated both. 30 The ad hoc resuscitation team subgroup had the highest proportion of high Kirkpatrick levels of evaluation, with both studies evaluating the effect of simulation on the behavior level (Level 3) and outcomes level (Level 4). In addition, the study by Andreatta et al was the only study included in this review that evaluated on all four Kirkpatrick levels. 29 Only four of seven studies in this review evaluated on the outcomes level (Level 4). 15 19 29 30 A summary of evaluation characteristics can be found in Tables 3 and 4 .

## Discussion

### In Situ Simulation versus Off Site Simulation Experiences

The majority (11 of 16 studies) developed in situ simulation programs rather than utilizing a designated simulation center. 17 18 19 21 22 23 25 27 28 29 30 Simulation in the real work setting has been identified as particularly valuable because it brings together all the elements of the care team and the environment. 30 In situ simulation therefore facilitates observation of the delivery of care as it happens, rather than how we speculate it may happen or as it should happen if didactic tools were to be followed precisely. 31

The value of in situ simulation was particularly well illustrated by studies seeking to identify system-level issues. Patterson et al acknowledged the role of in situ simulation in the training and evaluation of technical and nontechnical skills, but emphasized the unique ways in which the modality could be used to evaluate system competence and identify latent conditions that predispose to medical error. 18 This can be explained by the inherent overlap of in situ simulation and system-level evaluation in examining the conditions under which individuals work to build defenses and to avert or mitigate errors. 32 These authors conducted recurring in situ simulations to discover safety threats and system issues in this environment. The simulations served not only as a way to identify these issues but also as means of experiential learning that amplified the ability improve clinical processes. The authors noted that the in situ simulations prompted the identification of a latent threat in almost every simulation performed. They contrasted this rate of identification to that observed in the laboratory setting and attributed the difference to a more time-pressured environment and ability to test the actual clinical care system, including equipment, processes, and staff response. Beyond technical skills, Patterson et al noted that in situ simulation provided a means to continuously reinforce nontechnical skills (such as communication and teamwork skills).

### The Multidisciplinary Evolution of Teamwork Training

The literature on simulation in healthcare has gradually evolved from evaluations of individuals to evaluations of teams. 33 34 35 36 37 A similar progression occurred as early studies assessing specific skills performed by individuals were followed by studies evaluating nontechnical skills and teamwork. As the emphasis on teamwork increased, the value of incorporating team members from multiple disciplines in simulation activities became evident. In a review of high-fidelity simulation in critical care training by Boling and Hardin-Pierce, only 3 of 17 included articles were categorized as having a “mixed” population, in comparison to the remaining 14 articles with homogeneous populations of either nurses or physicians. 5 However, the importance of multidisciplinary teamwork is now better reflected in the participants of simulation literature, as illustrated by 14 of 16 included articles in this review assessing teams of multidisciplinary participants.

Some studies adopted a multidisciplinary approach at the earliest possible stages of their intervention, consulting with multiple stakeholders of the clinical team to refine the learning objectives and simulated scenarios. Barbeito et al conducted several interviews with residents, intensive care unit staff, members of their critical care committee and hospital leadership, critical care unit nurses and nursing aids, and other personnel. 30 This direct involvement across disciplines was of great benefit to the goals of the study, as participants were invested in the process of organizational change and felt encouraged to communicate system-level issues. The authors noted that some of the issues identified in debriefings were already well known to providers, and the program simply facilitated a formal way in which solutions could be implemented. For example, several experienced nurses had noticed occasional delays in establishing intravenous access during resuscitations. Protocols for the use of intraosseous devices were tested and refined during simulation and later implemented in actual codes.

Patterson et al also recognized the value of multidisciplinary training and discussion of social dynamics in the identification of latent safety threats at the system level. 18 These entities were described as system-based threats to patient safety that can materialize at any time and are previously unrecognized by healthcare providers, unit directors, or hospital administration. 38 Many of these previously unrecognized issues were brought to the attention of physician staff by nursing staff and prompted multidisciplinary problem solving. This collaboration catalyzed a shift in culture that emphasized safety and broke down implicit authority gradients. This paradigm shift was tested systematically by the purposeful addition of “mistakes” in multiple domains to simulations. During debriefings, discussion centered around times when team members did not feel comfortable addressing these issues despite knowing mistakes were being performed. Several team members described feeling an authority gradient during resuscitations, and this issue was addressed from a multidisciplinary standpoint in the same manner as other latent safety threats. This study emphasizes the role of the debriefing process not only in skill-based improvement, but also in the acquisition of a shared mental model, one of the focal concepts of quality improvement efforts. 16 39

Recent studies have continued to address the gaps in previous training platforms by modifying the culture to emphasize teamwork and expanding the scope of participants to reflect the importance of multidisciplinary training. 18 21 This paradigm shift is demonstrated by the structure and learning objectives of the Surgical Trauma Training Course (S2T2C). 21 This curriculum was developed to fill the gaps of traditional training by adhering to a team-based educational approach. This model included all personnel from corpsmen to surgeons participating in United States Navy pre-deployment training and demonstrated an emphasis on multidisciplinary team training. This emphasis reflects recent discussions of sociological fidelity in the context of simulation, which describes the interactions between learners as a means of creating authenticity and social realism. 40 41 As multidisciplinary team training is better characterized in the literature, there is more discussion of simulation as an opportunity to discuss social dynamics, hierarchy, power relations, and other factors affecting inter-professional teamwork. 15 16 42 43

### Evaluation of Effect on Patient Outcomes

The underlying motivation of the authors of each study in designing the simulation training programs was ultimately to improve patient outcomes. However, the majority of authors acknowledged that this goal was beyond the scope of their study objectives and/or would require substantially more statistical power to demonstrate. Aside from the four studies that attempted to evaluate patient outcomes, 15 19 29 30 all authors ended with a discussion of ways in which future research could expand upon their findings to assess actual patient impact. Since only four studies attempted to correlate their findings with institutional patient outcomes, only these four studies were identified as Level 4 evaluations in accordance with Kirkpatrick's levels of evaluation in Table 3 . 13 Two of these studies conducted simulations of the trauma bay, 15 19 and the remaining two studies conducted simulations of inpatient codes requiring ad hoc resuscitation teams. 29 30 Given the cost and administrative burden of developing simulation programs, these data are becoming increasingly important to support the experimental data already published.

The studies that assessed patient outcomes used a variety of different outcome measures reflecting the unique clinical environment of each acute care setting. Some studies have drawn indirect conclusions by monitoring clinical parameters before, during, and/or after the simulation and comparing outcomes. Andreatta et al used institutional pediatric CPA survival rates as a metric for patient outcomes and compared these rates to matched national averages. 19 29 The institutional survival rate also served as a proxy for the effectiveness of the intervention, as they compared these data longitudinally before and after the simulation program. At the beginning of the study (when only 10 “informal mock codes” had been run), the pediatric CPA rate was 33%. However, they observed a significant increase to ∼50% within 1 year, which also correlated temporally with increased frequency of mock codes when plotted. The authors also compared survival rates by the type of arrhythmia triggering the code and noted the improvements correlated with the times those rhythms were being simulated in mock code scenarios. This information was interpreted as evidence that the content being taught in the simulations was translating to improved patient outcomes.

Steinemann et al also analyzed clinical process parameters, including time to completion and reporting of key elements of the primary trauma survey, focused abdominal ultrasound, times in and out of the ER, number and type of procedures performed, units of blood transfused, and delays to patient transfer. 19 Corresponding patient data were also recorded, such as gender, morbidity, mortality, and length of stay. While the authors observed significant improvements in mean teamwork scores and objective parameters, such as speed and completeness of resuscitation, no significant change was noted in global clinical endpoints, such as mortality, morbidity, or length of stay. The study therefore observed significant differences in Level 3 evaluations (translation of learning to clinical setting as illustrated by improved objective parameters), but did not observe significant differences in Level 4 evaluations (patient outcomes). 13

Other studies assessed potential impact on patient outcomes more qualitatively by continuously evaluating the system-level effect of the simulation program. 15 30 Barbeito et al achieved this by designing the intervention in a reiterative way to both identify system-level issues and to assess attempts at resolving these issues over time. 30 This facilitated an indirect assessment of patient outcomes through monitoring the consequences of process changes. For example, debriefings revealed inefficiency in the way samples were collected, and laboratory studies were ordered during mock codes. This issue was addressed by the creation of a standard set of laboratories (“code labs”) that were then automatically ordered during codes. This process change was then introduced into actual codes, and a decreased incidence of laboratory order entry errors was observed after implementation of the new workflow. Capella et al reported a similar pattern of results in their Level 3 and Level 4 evaluations, although their experimental setup was different. 15 The authors observed significantly decreased times to task completion (times from arrival to computed tomography [CT] scanner, endotracheal intubation, and operating room), but patient outcome data were not significantly different between the two groups (intensive care unit length of stay, hospital length of stay, complication rate, and mortality rate). Both studies cited a small sample size as the principle reason for not observing significant differences in patient outcomes given the changes in objective parameters observed. 15 30

### Critique of Current Evidence

There are very few randomized studies of simulation in team training, particularly in the acute care setting. Only one of the included articles in this review randomized participants. 25 Gundrosen et al randomized nurses that were being introduced to a new clinical guideline via either lecture-based or simulation-based teaching methods and evaluated the effect on non-technical skills. 25 Another common limitation of the current simulation literature is the lack of a control group. It is therefore challenging to rule out whether the effects seen were due to chance or characteristics specific to the participants in the intervention group. Some studies have attempted to address this by using a pre- and post-intervention design where the study group served as their own control. 20 21 The S2T2C used a prospective observational study design where the participants served as their own controls. 21 Similarly, Acero et al used a “cold” simulation to evaluate participants' baseline knowledge and skills, later comparing these results to a “warm” simulation after formal training had been given. 20 Other groups have instead discussed why a static control and experimental arm is not feasible or desirable in the trauma setting, since in reality, trauma groups change composition dynamically. 17 As another alternative, some studies did not utilize control groups, but instead intentionally formed other comparable groups. 26 For example, Pascual et al validated their intensive care curriculum for advanced practitioners by having recently graduated critical care fellows participate and serve as the “gold standard comparison group.” 26

Very few studies evaluated whether the observed effects of the interventions were sustainable. Those studies that assessed sustainability had mixed findings. Miller et al conducted in situ simulations with the goal of improving teamwork and communication skills in the trauma setting. 17 The authors evaluated the effect of the simulations in several phases, including a “potential decay phase” in which sustainability was assessed after the simulation training had ended. All observed benefits had declined, and the authors concluded that, while an in situ simulation program can be effective in improving teamwork and communication in the clinical setting, these benefits are lost if the simulation program is not continued. Hoang et al evaluated technical and nontechnical skills in simulated combat via the S2T2C at different points in time. 21 In contrast, the authors observed improved teamwork and communication skills upon the completion of the S2T2C as well as after 5 months had passed. However, despite sustainment of significantly improved disposition times 5 months later, these times did increase, indicating the necessity of refresher courses to optimize training outcomes. Other studies evaluated sustainability indirectly, through clinical outcome measures assessed after the simulation period had ended.

Sustainment was also assessed through longitudinal study design. 28 Assessing the impact of an embedded simulation team training program in a pediatric intensive care unit, Stocker et al observed a 6- to 12-month learning curve. 28 The authors concluded that repeated exposure to simulation is the most beneficial in crisis resource management training, and single, isolated exposure may not be sufficient. However, a limitation of this study was the use of participant self-reporting to assess effectiveness.

### Need for Future Research

Although the quantity of simulation-based research has continued to increase steadily, the quality is highly variable, and further research is sorely needed. 44 45 A major barrier to further implementation of simulation in acute care training is the associated cost. Very few studies provide information on the costs of initiating and running simulation programs, but this information is necessary for the justification of this investment in an era of tightening healthcare budgets. For example, Acero et al reported a cost of $3.8 million in the building of their institution's simulation center and an additional $1.5 million annually for associated operating expenses. 20 Other financial and administrative burdens requiring further investigation include the opportunity costs of removing participants from clinical duties and/or occupying clinical areas (especially operating rooms) for in situ simulations. Finally, as previously discussed, randomized studies with larger sample sizes and the statistical power to evaluate the impact of simulations on actual patient outcomes are necessary. Data illustrating statistically significant changes in patient outcomes, such as length of stay, would enable more sophisticated cost analyses exploring the utility of wider implementation of simulation programs. More research on the sustainability of these outcomes will also be necessary to model the future impact of these investments.

### Study Limitations

This study is not without limitations. Although the search process was rigorous, it is still possible that some relevant studies were missed and therefore not included in this review. The predefined search strategy may have left out keywords that would have potentially captured additional relevant studies. For example, the use of the predefined terms “surgery,” “trauma,” and “critical care” and the subsequent application of predefined inclusion and exclusion criteria did not yield obstetrics and gynecology simulation research, although simulated acute care scenarios may be created in this context. In addition, the review synthesized a relatively small amount of studies that each had relatively small sample sizes. This potentially limits the strength and generalizability of conclusions and the accurate identification of themes. However, the number of studies included is comparable with integrative reviews of similar scope such as Boling `and Hardin-Pierce (17 studies), 5 Gjerra et al (13 studies), 3 and Warren et al (10 studies). 10

## Conclusions

High-fidelity team-based simulation is feasible in a wide variety of acute care settings, including emergency departments/trauma bays, operating rooms, intensive care units of multiple types, and inpatient ad hoc resuscitation teams. It is an effective means of training and/or evaluating multidisciplinary teams in both technical and nontechnical skills and has the capacity to facilitate organizational- and system-level change. It is also a way of involving the input of multiple stakeholders and can improve multidisciplinary teamwork. Studies over the last 10 years have been heterogeneous in both intervention and evaluation design, and there is still a paucity of validated instruments available for this context. However, a more standardized approach to team-based simulation is necessary to generate generalizable conclusions and to provide evidence-based guidance for future simulation planning. These conclusions could also enhance the role of low- and medium-fidelity team-based simulation in environments where high-fidelity simulation is not possible due to logistic or financial reasons. As our understanding of “psychological fidelity” improves, the elements most critical to developing multidisciplinary teamwork skills can be reproduced in lower fidelity simulations, such as task trainers, computer-based systems, and virtual reality systems. Finally, there are currently no studies that have demonstrated significant improvements in patient outcome metrics, such as mortality or length of stay. The impact on patient outcomes and the sustainability of simulation efforts are areas that warrant further research.
